# The juvenile social environment introduces variation in the choice and expression of sexually selected traits

**DOI:** 10.1002/ece3.230

**Published:** 2012-05

**Authors:** Michael M Kasumovic, Matthew D Hall, Robert C Brooks

**Affiliations:** 1 Evolution & Ecology Research Centre, School of Biological, Earth & Environmental Sciences, The University of New South WalesKensington, Sydney 2052 NSW, Australia; 2 Zoologisches Institut, University of BaselBasel CH-4051, Switzerland

**Keywords:** Adult behavior, age-specific calling effort, condition dependence, developmental plasticity, juvenile environment, social environment

## Abstract

The juvenile environment provides numerous cues of the intensity of competition and the availability of mates in the near environment. As research demonstrates that the developing individuals can use these cues to alter their developmental trajectories, and therefore, adult phenotypes, we examined whether social cues available during development can affect the expression and the preference of sexually selected traits. To examine this, we used the Australian black field cricket (*Telogryllus commodus*), a species where condition at maturity is known to affect both male calling effort and female choice. We mimicked different social environments by rearing juveniles in two different densities crossed with three different calling environments. We demonstrate that the social environment affected female response speed but not preference, and male age-specific calling effort (especially the rate of senescence in calling effort) but not the structural/temporal parameters of calls. These results demonstrate that the social environment can introduce variation in sexually selected traits by modifying the behavioral components of male production and female choice, suggesting that the social environment may be an overlooked source of phenotypic variation. We discuss the plasticity of trait expression and preference in reference to estimations of male quality and the concept of condition dependence.

## Introduction

The origin and nature of variation in sexually selected traits and mate choice is of considerable theoretic and practical interest because this is the variation on which mate choice is based (Kirkpatrick and Ryan 1991; Iwasa and Pomiankowski 1995; Kotiaho et al. 2001; Kokko et al. 2003). Not all aspects of choice and signaling, however, should be equally variable or equally plastic. An understanding of the nature of variation in the various aspects of a mating system is important if we are to understand which of the constituent traits respond to environmental variation and how females respond to such variation. Many structural and spectral properties of acoustic, olfactory, and color signals are under stabilizing selection because receptor organs are tuned to particular frequencies, chemicals, wavelengths, and temporal structures. For example, the central auditory system of female cricket frogs (*Acris crepitans*) is most sensitive to an intermediate frequency within the range of call frequencies made by males (Ryan and Wilczynski 1988), and this exerts stabilizing sexual selection on male call frequency. A variety of other studies document preferences for intermediate values of call frequency and various measures of temporal structure in the acoustic calls of frogs (Ryan and Keddy-Hector 1992; Polakow et al. 1995; Murphy and Gerhardt 2000) and insects (Ritchie 1996; Wagner 1998). In contrast, aspects of male's signals that involve differential energetic investment such as in the intensity or duration of signaling should harbor more variation as males vary in their ability to invest into such traits (Rowe and Houle 1996) and because signaling can also come at an ecological cost (Ryan 1988). Dissecting how the environment affects variation in sexually selected traits therefore needs to consider both the shifts in female choice and the type of selection that each of the traits that comprise a male's signal experience.

To that end, a large proportion of the sexual selection literature is devoted to examining how choice and courtship behavior is altered by an individual's internal state or condition as a function of resource acquisition during either the juvenile or adult stage. Following the suggestion by Andersson (1982) that condition dependence could maintain variance in sexually selected traits, and by Rowe & Houle's (1996) formalization of the idea that condition-dependent traits could “capture” the ever-shifting pool of genome-wide variance underlying condition, there has been an avalanche of papers on condition-dependent courtship and ornamentation (Cotton et al. 2004; Tomkins et al. 2004; Bussière et al. 2008; Johnstone et al. 2009). In addition, condition is also known to affect female choice (Hunt et al. 2005; Cotton et al. 2006). Condition dependence, however, is a notion of developmental plasticity where the environment is specifically limited to examinations of resource (or nutrient) acquisition. Environments, however, vary in more than resource abundance and expanding the environment to include a greater variety of environmental triggers that are known to alter allocation strategies would provide insight in the variation in trait expression and choice.

One such factor is the social environment. The social environment is known to alter the intensity and direction of selection as a function of the number of rivals and the availability of mates (Kasumovic et al. 2008; Punzalan et al. 2010) and is an important factor in determining trait expression (socially cued anticipatory plasticity; Kasumovic and Brooks 2011). As a result, it is likely an understudied factor in determining trait expression and choice. The juvenile environment is known to affect resource allocation patterns during juvenile development (Kasumovic and Brooks 2011) resulting in shifts in phenotypic expression at maturity (e.g., Kasumovic and Andrade 2006; Kasumovic et al. 2011). There are also recent results that suggest that the juvenile environment can have an influence on the evolution of sexually selected traits. For example, from a male's perspective, shifts in the competitive environment can increase the cost of signaling (Shine et al. 2003; Clark and Grant 2010) and therefore, male signaling effort. The juvenile environment could signal the type of competitive challenges males will encounter and males in turn could alter their behavioral strategies (Bailey et al. 2010), resulting in a shift in signal expression (Kasumovic et al. 2011).

In a similar manner, the juvenile environment could signal the quality and density of mates available and females could alter their choice. How the juvenile environment affects female choice is slightly more complex as variation in choice is best understood by decomposing it into estimates of preference and response speed (Jennions and Petrie 1997). Preference (or preference function) refers to the relationship between the properties of possible mates and an individual's behavior toward those individuals (or proxy stimuli) while response speed describes how quickly females respond to a mate or appropriate stimulus. Environmental factors that affect choice can thus do so by affecting either a female's preference and/or response speed. For example, the environment experienced while immature can alter female choice by either affecting preference for an experienced or unexperienced phenotype (e.g., Hebets 2003; Rutledge et al. 2010) or response speed when making decisions about the “quality” of a signal (e.g., calling rate; Bailey and Zuk 2008).

Although the social environment remains an understudied potential contributor of variance in sexually selected traits, there is the potential for the juvenile environment to affect the expression and evolution of sexually selected traits from the perspective of both sexes. The relative importance of the social environment, however, remains unknown as the few studies that have examined the effect of the juvenile social environment on the expression of sexually selected traits have focused on a single sex in absence of the opposite sex (e.g., Hebets 2003; Rutledge et al. 2010) and examine traits under directional selection (e.g., Bailey and Zuk 2008). To truly understand the relative importance of the juvenile environment requires a simultaneous examination of both sexes and the effect of the juvenile environment on the expression and choice of both sexually and nonsexually selected traits. This will provide the much needed insight into the capacity of the juvenile social environment in shaping the maintenance of expression sexually selected traits and trait evolution in general.

**Figure 1:**
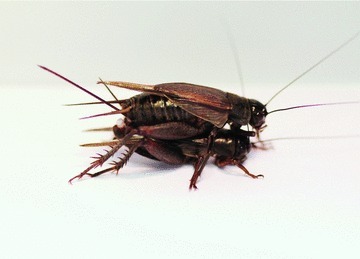
A pair of mating black field crickets (*Teleogryllus commodus*). The male is on the bottom and has produced a spermatophore that he is attempting to attach to the female sitting on top. Photo credit: Edith Aloise-King.

To examine whether the social environment experienced during development can contribute to variation in male sexually selected traits and female choice and to determine the extent to which shifts are correlated, we used the black field cricket (*Teleogryllus commodus*; Fig. 1); a species where individual condition affects both female choice through response speed (Hunt et al. 2005) and male signaling effort (Hunt et al. 2004). Male *T. commodus* broadcast a long-distance advertisement call to attract potential mates (Evans 1988). The call dominant frequency (DF) and temporal structure of the pulses that make up the chirp and trills are under stabilizing selection (Brooks et al. 2005; Bentsen et al. 2006). Calling effort, however, is under strong directional sexual selection. In fact the multivariate selection gradient that best aligns with calling effort also has a positive quadratic component; in the wild, all calls with below average calling effort are equally unattractive, and then there is a nonlinear increase in attractiveness with increasing calling effort (Bentsen et al. 2006). Calling effort is both energetically and ecologically costly (Ryan 1988; Hoback and Wagner 1997) such that only males in relatively better condition can afford the costs associated with calling (Hunt et al. 2004). Furthermore, calling also attracts rival males, possibly satellites (Bentsen et al. 2006), such that high calling effort also elevates intrasexual selection. Individual calling rate is thus most often used as an indicator of male quality in *T. commodus*. Most importantly however, the social environment significantly affects juvenile allocation patterns (Kasumovic et al. 2011); males mature more quickly and at a smaller size when they perceive less competition, while females mature more quickly and at a smaller size when they perceive a greater density of males calling at a higher rate.

Here we examine whether individuals alter the expression and choice of sexually selected traits in response to the rate and density of male calls they hear as juveniles. As calling effort (i.e., rate) is under directional selection, we use calling effort as a surrogate for quality with males calling at a higher rate being considered of higher quality. We reared juvenile *T. commodus* males and females in social environments that varied in the density and rate of male calls. In two of the treatments, we manipulated the calling rate of the calls we broadcast to immature individuals by playing all calls at either a low or a high calling rate simulating populations of males comprising calling at low and high rates. In the third calling treatment we played calls at three different rates, simulating a population where males vary in their calling rate. We crossed the three calling treatments with two different densities where we altered cues of adult male density by broadcasting calls in one treatment at four times the density of the other. Using this experimental design, we examined whether the juvenile social environment as determined by the density and rate of calls affected the female preference and/or response speed, as well as the structural properties of male's calls and male age-dependent calling effort. For females, we predict that the social environment will alter female response speed rather than preference just as seen by variation in condition (Hunt et al. 2005). For males, we predict that call properties known to be under stabilizing selection should not be highly susceptible to variation in the social environment, while calling effort will be far more likely to show variation in response to the social environment as it is under directional selection.

## Methods

Crickets used in this experiment were third generation descendents of approximately 100 females collected at Smith's Lake (32°22′S, 152°30′E), New South Wales, Australia, in March 2008. At the start of the experiment, we collected 250 nymphs within 24 h of hatching and three weeks later collected 250 more nymphs to separate the time to eclosion and facilitate adult measurements. Each nymph was reared in an individual plastic container (5 × 5 × 3 cm) with an egg carton for shelter and supplied with ad libitum food (Friskies Go-Cat senior) and water. Water and food were replaced weekly. Individuals were randomly assigned in one of six experimental calling treatments (see below) and checked daily for adult eclosion.

### Experimental treatments

For this experiment, we manufactured calls using the natural calls of four different males randomly selected from the stock population. Using Adobe Audition (version 3.0), we manipulated the intercall duration (ICD) according to the variation outlined in Hunt et al. (2005). We only manipulated ICD as female preference on ICD is linear, resulting on selection for shorter intercall duration. Briefly, we used two calls from one male and manipulated the ICD to create a calling bout with a mean ICD (according to Hunt et al. 2005). We then manipulated the ICD to create six different calling bouts that varied—1 (highest call rate), 1, 2, 3, 4, 5 (lowest call rate) standard deviations (SDs) from the mean ICD. These calls were used for the female choice trials (see below).We then used two calls from each of the three remaining males to create a calling bout with a mean ICD and then altered the ICD within these calling bouts—1 and 5 SDs to create the high (25 calls/min) and low (12.5 calls/min) calling rates treatments, respectively. We used one call from each of the three males in each of the treatments (see below) to ensure that all developing individuals heard the same three males.

We mimicked different social environments by altering the rate and the density of calling males; each coming from a single speaker in a different direction (see below). To vary the rate of calling males, we reared females in treatments with (1) only low calling rates (5 SD), (2) only high calling rates (–1 SD), and (3) variation in calling rates (5, 0, –1 SD). Given the natural variation in ICD, the calls would be naturally in and out of synch throughout the evening. To vary social density, we reared individuals in either a (1) high density (12 calling males) or (2) low density (three calling males) environment. Since our high-density trials had four replicates of the same three males calling, we controlled for the number of different calling males and any differences found due to our density treatment would be due to an increased number of males calling, rather than an increased variation of calling males. This resulted in a total of six different treatments where the treatments differed in only density and rate of the calls as all the individuals experienced the same three males’ calls during development.

Due to the difficulty in acoustically isolating each treatment, we set up six different acoustically isolated environments consisting of different rooms. Each treatment was kept in a different room under the same temperature, light cycle and calling cycle. We randomly moved each treatment to a different room each day to ensure no room effects. In each room, we placed 12 speakers (Logitech R-10) in a 1-m diameter circle and we ensured that all speakers played calls at an amplitude of 70 dB at 50 cm. We played male's calls in.WAV format using mp3 players (Sandisk sansa c240 1GB) with Rockbox firmware (http://www.rockbox.org/). Individuals were stacked within the center of each speaker arrangement and placement of individuals was randomized during each movement.

### Female choice

Upon maturity, we kept females in a common room in acoustic isolation until day 10 when they were tested in the mate choice arena to determine their choice by examining their preference and response speed. We used a 106 × 106 cm choice arena where speakers were placed at 180° flush with the arena wall. We broadcast calls at an amplitude of 70 dB in the center of the arena. For each trial, we placed a female in the center of the arena under a plastic perforated 4-cm diameter cylinder. A focal call was played against a control call (mean, 0 SD) for 1 min, after which the cylinder was removed and the female was allowed to make a choice. We considered a choice of either the focal or control call if the female came within a 6.5-cm radius of a speaker. We recorded the preferred call and the amount of time it took females to make a choice. All females made a choice within 5 min.

Each female was used in six consecutive choice trials (for a total of 966 trials) in each of which one of the focal calls was played against a control call. We recorded the preference and the time to make the choice (i.e., response speed) for each trial. Both call order and speaker presentation was randomized for each trial. Trials were completed under red light to minimize observer disturbance. We scored a female's preference as 0 (preference for the standard call) or 1 (preference for the focal call).

### Male life span and calling effort

Upon maturity, we placed males in a custom-built electronic monitoring device (see Hunt et al. 2004) overnight every three days until death to determine age-specific calling effort. Briefly, the device consists of 64 microphones attached to the lids of male containers that are connected to the sensor; the sensor is connected to a DaqBook 120, IO Tech data logger, and personal computer, which is programed to check for signal from each microphone 10 times per second. The signal is recorded as 1 when 10 dB higher than the level of background noise, otherwise as 0. Males were kept in individual containers (5 × 5 × 3 cm), which were then placed in plastic containers (14 × 6 × 6 cm) surrounded by acoustic foam to keep males in acoustic isolation. We measured calling effort of each male for 12 h every third day and calculated an average daily calling rate for each male. When calling effort was not measured, males were kept within a common room with stock males and therefore heard variation in calling effort throughout their adult life span.

### Call structure analysis

We recorded the calls of males as uncompressed audio in an acoustically isolated room at approximately 22°C using a Sony Hi-MD walkman (MZ-NH700, Sony, Japan). The recorder was attached to a condenser microphone (C1163, Dick Smith Electronics, Australia), which was mounted in the lid of the cricket containers. To power the microphone a custom junction unit was used between the microphone and recorder. We used Raven sound analysis software (version 1.2, Cornell Bioacoustics Research Program, Ithaca, NY) to measure chirp pulse number (CPN), trill number (TN), intercall duration (ICD), chirp pulse duration (CIPD), and DF from five randomly selected calls per male.

### Statistical analysis

We used a two-way analysis of variance (ANOVA) to examine whether the rearing treatment (density and call rate) had an effect on life span. We used a two-way multivariate analysis of variance (MANOVA) to examine whether the rearing treatment had an effect on call parameters. We log transformed TN, ICD, and CIPD. We also log transformed both daily calling effort and female response speed in the choice trials for the analyses below.

To examine the effect of rearing treatment on female preference and response speed, we again used a mixed-model approach to examine the effect of juvenile experience (density and call rate) on preference and response speed separately, with female identity as a random factor. Since the order of presentation of the different choice trials was randomized, we factored in the order of presentation and the focal call as covariates within the model. We then added focal call by rearing treatment interactions. A significant order effect would signify that the experience of hearing previous male's calls altered a female's choice. A significant focal call would signify that a female's choice depended on the difference between the standard and focal call.

To examine how calling effort changed with age and the various juvenile acoustic treatments, we used a mixed-model approach to fit a series of multiple regression models to the data. Essentially this process is an multivariate extension of a two factor analysis of covariance (ANCOVA; e.g., Crawley 2008; p. 500), where each regression modeled the relationship between calling effort and age as a second-order polynomial regression (age and age^2^) with the treatments as additional fixed effects and male identity as a random effect. We began with the quadratic model as previous studies have shown that age-specific calling follows a nonlinear trend (Hunt et al. 2004; Maklakov et al. 2009; Zajitschek et al. 2009b). The series of regression models, therefore, represent specific hypotheses regarding how the treatments potentially influenced male calling effort, ranging from a single pattern of age-specific calling investment for all treatments (no interaction terms included), to separate patterns for every combination of density and calling rate (including three-way interaction terms).

To distinguish between the different candidate regression models we used Akaike information criteria (AIC) to assess how well each model describes the data (Akaike 1983), with smaller values representing a better fit. The resulting AIC values were then used to rank the evaluated models. In general, only models that differ by two or more AIC units provide distinguishable levels of support (Burnham and Anderson 2002). We also calculated the AIC weights for each model, which provides a relative weight of evidence for given model in comparison to all other candidate models (Burnham and Anderson 2002). We then calculated the linear and quadratic parameter estimates for the calling curves for each of the relevant treatments to determine where exactly the differences in calling effort occur. All statistics were performed in R (version 2.9.2, R Development Core Team, www.R-project.org) using mixed-model analyses as implemented with the lme4 package (Bates et al. 2008). Age data used for this analysis was first restricted to records up to and including 70 days posteclosion to avoid a potential bias arising from small sample sizes at the later age points, and then standardized to a mean of zero and SD of one. Finally, we visualized the nonlinear trends of the best-fitting regression model using nonparametric splines generated with the general additive mixed-model package (gamm4, Wood 2009) of R.

## Results

This experiment was part of a larger study examining the effect of the juvenile social environment on development (Kasumovic et al. 2011). Of the 338 individuals that successfully reached maturity from the development experiment, we collected lifetime calling information for 151 males (high density: 24 low, 23 high, and 25 variable call rate; low density: 25 low, 25 high, and 29 variable call rate), and examined the call structure for 124 males. We examined the preference of 161 females (high density: 23 low, 30 high, and 29 variable call rate; low density: 25 low, 25 high, and 29 variable call rate).

### Female choice

**Figure 2:**
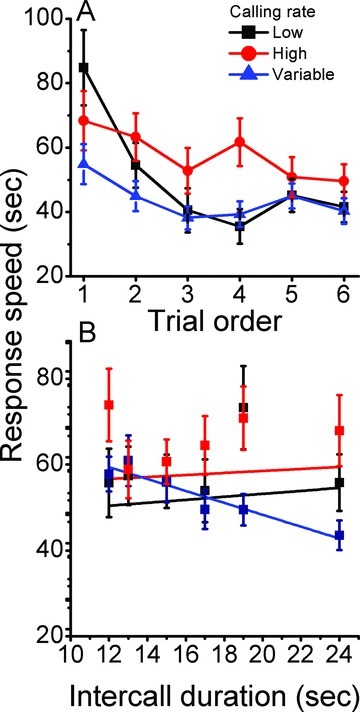
The average response speed of females from each calling rate treatment (different colors). Panel A indicates how a female's response speed changes after each subsequent trial. Panel B indicates how quickly females chose a speaker in a trial when presented with a control call against the focal call with a specific intercall duration (*x*-axis). A shorter intercall duration indicates a faster calling rate. Bars are standard errors.

All females preferred calls with a lower ICD (*F*_1, 798_= 42.06, *P* < 0.0001). There were no other significant effects on the preference of females (all *P* > 0.05). A female's response speed, however, was affected by two factors. First, the order of presentation of the calls affected response speed with females responding more quickly in later trials (Table 1, Fig. 2A). Second, there was a significant calling rate × focal call interaction (Table 1) with females reared in the variable calling rate treatment discriminating between lower calling rates and the standard more quickly than individuals reared in the high and low calling rate treatments (Fig. 2B).

**Table 1:** Results from a mixed model examining the effect of rearing density, calling rate, the order of presentation, and the focal call used in the choice trial on the response speed of females.

	*F*	df	*P*
Density	0.62	1, 155	0.80
Calling rate	0.12	2, 155	0.09
Density × Calling rate	0.19	2, 155	0.86
Order	0.89	5, 794	<0.0001
Focal call	41.80	1, 794	0.09
Density × Focal call	1.27	2, 794	0.72
Calling rate × Focal call	1.34	2, 794	0.039
Density × Calling rate × Focal call	0.89	5, 794	0.99

### Male life span and calling effort

We used a two-way ANOVA to determine whether life span was affected by the social environment. There was a significant effect of density on life span (*F*_1,148_= 4.28, *P*= 0.04) with males in the lower density treatments living longer (low density: 46.31 ± 1.92, high density: 40.59 ± 2.00). There was no effect of calling rate (*F*_2,148_= 0.25, *P*= 0.78) or a density by calling rate interaction (*F*_2,148_= 0.44, *P*= 0.64).

We also assessed if the juvenile acoustic environment influenced the changes in male calling effort that occur with increasing age. In total we evaluated six models (Table 2), beginning with the intercept only model (model 1), where only the experimental factors (density and calling rate treatments) are fitted and there is no relationship between age and calling effort. We then fitted age, age^2^, and various interactions between the experimental factors and these age-dependent covariates until we reached the most complex model (model 6) involving three-way interactions between density, calling rate, and age-specific calling effort. Overall this model (model 6) best described the relationship between age-specific calling and the experimental acoustic environments.

**Table 2:** The six-candidate regression models describing how patterns of age-specific investment in calling effort depend on the juvenile acoustic environment that males experience. The models are listed in order of complexity, beginning with a null model where no age-specific patterns of calling investment were estimated (model 1), and ending with the most complex model where separate patterns were estimated for every combination of density and calling rate treatments (model 6). Presented for each model are the corresponding AIC scores and the AIC weights, where larger values indicate greater relative support for the given model in comparison to all other candidate models.

Candidate models for patterns of age-specific calling effort	Terms added	Total factors	AIC	AIC weight
(1) No aging curve	Treatment intercepts	0	15,249	<0.001
(2) Single curve for all treatments	Age	2	14,169	<0.001
	Age^2^			
(3) Different curves for density treatments only	Density × Age	5	14,174	<0.001
	Density × Age^2^			
(4) Different curves for calling rate treatments only	Calling rate × Age	5	14,138	0.067
	Calling rate × Age^2^			
(5) Different curves for calling rate and density treatments independently	Density × Age	8	14,137	0.111
	Calling rate × Age			
	Density × Age^2^			
	Calling rate × Age^2^			
(6) Different curves for every density and calling rate combination	Density × Calling rate × Age	11	14,133	0.821
	Density × Calling rate × Age^2^			

**Figure 3:**
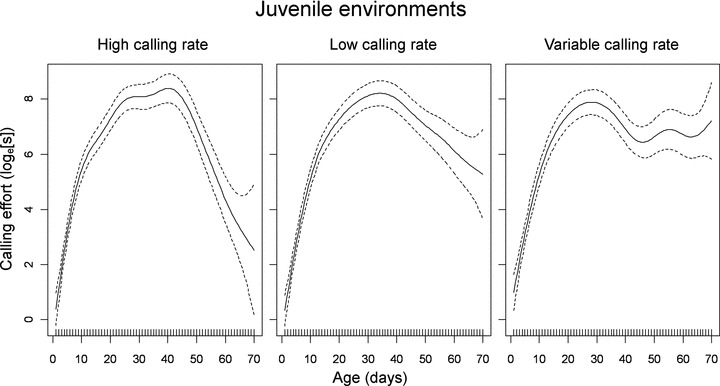
The average age-specific calling effort of males reared in the high, low, and variable calling rate treatments. Curves were visualized with a best-fitting regression model using nonparametric splines generated with the general additive mixed-model package (gamm4). Dashed lines are 95% confidence intervals.

The effects of our treatments on age-dependent calling patterns appear to be due to differences in what happens after males reach peak calling at about 30 days of age. Reanalyzing only the calling effort data for males of 30 days or younger (in the same way we analyzed the data for Table 2) revealed no differences among treatments in age-specific calling effort (all *P* > 0.22). The linear and quadratic coefficients for all treatment combinations (Table 3) reveal the importance of differences in the rate at which calling effort senesced for each of the calling curves. The quadratic terms are more strongly negative (i.e., a stronger convex pattern) for males from the higher calling rate environments, followed by the low calling rate and then the variable calling rate environments. More strongly negative quadratic coefficients in the high density and high calling rate treatments arise because of the rapid senescence in calling effort among males from these treatments (Fig. 3) than in other treatments.

**Table 3:** The standardized regression coefficients describing patterns of age-specific calling effort (the calling curves) for each treatment estimated using a single regression for each treatment (density and calling rate) combination. The individual parameters allow comparison of the linear (age) and quadratic (age^2^) estimates between the different treatments, demonstrating a more rapid senescence of calling effort in the high versus the low and variable calling rate treatments.

Density	Calling rate	Linear terms			Quadratic terms		
		Estimate	SE	*P*-value	Estimate	SE	*P*-value
High	High	7.857	0.700	<0.001	–7.513	0.856	<0.001
	Low	8.063	0.482	<0.001	–6.877	0.536	<0.001
	Variable	6.460	0.555	<0.001	–5.252	0.565	<0.001
Low	High	8.462	0.427	<0.001	–7.517	0.430	<0.001
	Low	6.974	0.471	<0.001	–5.747	0.471	<0.001
	Variable	4.363	0.524	<0.001	–3.202	0.486	<0.001

### Call structure

There was no significant effect of either density (*F*_5,114_= 0.31, *P*= 0.90) or calling rate (*F*_10,228_= 0.79, *P*= 0.64) on any call parameters. There was also no density × calling rate effect on call parameters (*F*_10,228_= 1.27, *P*= 0.25). The social environment thus had no effect on the variation in any structural or temporal properties of males’ calls (Table 4).

**Table 4:** The treatment means and standard errors for the advertisement call characteristics including chirp pulse number (CPN), chirp pulse duration (CIPD), trill number (TN), intercall duration (ICD), and dominant frequency (DF).

Density	Calling rate	CPN		CIPD (s)		TN		ICD (s)		DF (kHz)	
		Mean	SE	Mean	SE	Mean	SE	Mean	SE	Mean	SE
High	High	5.486	0.241	0.015	0.001	2.471	0.379	0.126	0.014	4.064	0.034
	Low	5.907	0.177	0.014	0.001	2.788	0.278	0.148	0.010	4.030	0.025
	Variable	6.267	0.202	0.014	0.001	3.060	0.317	0.160	0.012	4.085	0.028
Low	High	5.889	0.180	0.014	0.001	2.532	0.284	0.139	0.011	4.042	0.025
	Low	6.150	0.201	0.014	0.001	2.810	0.317	0.139	0.012	4.041	0.028
	Variable	5.557	0.207	0.015	0.001	2.642	0.325	0.155	0.012	4.048	0.029

## Discussion

Empiricists have spent considerable effort dissecting the variation in female choice (Iwasa and Pomiankowski 1995; Jennions and Petrie 1997; Cotton et al. 2006) and male sexual signals (Hunt et al. 2004; Petfield et al. 2005; Hall et al. 2010; Ingleby et al. 2010) and exploring how such variance in mate choice arises and persists despite strong sexual selection (Kirkpatrick and Ryan 1991; Andersson 1994; Rowe and Houle 1996; Tomkins et al. 2004; Bussière et al. 2008; Johnstone et al. 2009). Here we provide experimental evidence that in the black field cricket, *T. commodus*, the rate and quantity of male calls that individuals hear while immature significantly affects the behavioral aspects associated with phenotypic variation in both female and male sides of mate choice. The social environment experienced while immature affects how quickly females choose a mate (i.e., response speed) and how males call throughout their lifetime. Our findings have important implications for understanding which components of sexual selection will be most sensitive to the variation induced by the social environment.

### Variation in female choice

Variation in female choice can be a result of changes in preference and/or response speed (Jennions and Petrie 1997). We found no effect of the juvenile environment on mate preference in *T. commodus*, which agrees with data demonstrating strong directional selection for male calling effort in this species (Brooks et al. 2005; Bentsen et al. 2006). Although shifts in preference are rare, they do exist and are shown to vary with condition (Riebel et al. 2009) or with social experience as in a recent Internet-based study, DeBruine et al. (2010) even showed that women from different countries have different preference for facial masculinity. The social environment may not affect preference in *T. commodus* as variation in calling effort is continuous and unimodal and shifts in preference may be more common in species where there is bimodal distribution in phenotypes (e.g., Hebets and Vink 2007; Hebets et al. 2008; Rutledge et al. 2010) or multiple preference peaks due to multivariate ornamentation (e.g., Rosenqvist and Houde 1997; Blows et al. 2003).

We did, however, demonstrate variation in response speed. Response speed should, in most mating systems, be a more plastic trait than preference, allowing females to facultatively respond to their own condition, as well as the presence of predators and other risk factors in the environment (Hunt et al. 2004; Syriatowicz and Brooks 2004; Fox and Moya-Larano 2009; Woodgate et al. 2009), and accordingly adjust their willingness to be courted and to mate. Our results indicate that juvenile social environment too, in the form of the adult male calls an immature female hears, can influence female response speed in *T. commodus* as females that experienced a greater variation in calling rate as juveniles chose the speaker with a higher call rate more quickly when there was a greater difference in the call rates between speakers (variable calling rate treatment; Fig. 2B). Although this response difference diminished through time as a female gained greater experience after maturity (Fig. 2A), we have no information on how long this such experience is maintained and whether females need to “re-experience” the variance in calls for such an effect to be maintained as all trials for each female were completed within 30 min.

Regardless, our results demonstrate that the juvenile environment “primes” female response speed similar to the effects of diet manipulation on these traits in *T. commodus* (Hunt et al. 2005)—although females on high-quality diets responded more rapidly, there was only modest condition-dependent variation in which stimulus values females preferred. Our results are also consistent with the effects of the presence or absence of calls during development in a congener (*T. oceanicus*) where females take longer to discriminate when reared in the absence of calls (Bailey and Zuk 2008). Most importantly, however, our results demonstrate that changes in response speed can result from variation in the rates of calls heard while immature and not simply binomial extremes as in the presence/absence of a cue (Hebets and Vink 2007; Bailey and Zuk 2008) as has previously been the focus. Currently, the effects of such variance in response speed on sexual selection itself is relatively unexplored, however, shifts in response speed could result in different effects on selection depending on the availability of mates in the environment at different times of the season or the relative competitive ability of the cohort of males. Future studies are necessary to further examine this phenomenon.

### Variation in male sexual signals

Sexual signals often contain both relatively stable structural components (e.g., color properties, fine-scale acoustic spectra and timing properties, particular combinations of chemical components) as well as more plastic components that often vary in magnitude (e.g., rates of courtship display, size of ornaments, volume or duration of calls, or amount of pheromone released). The former traits are more often under stabilizing sexual selection than the latter, especially if the sense organs of potential mates are tuned to particular combinations of these properties, making deviant signals less effective (e.g., Ryan and Wilczynski 1988; Ryan et al. 1992). These are often the signal properties most heavily used in recognizing potential mates (Ryan 1990; Gerhardt 1991) and are less influenced by other environmental factors such as diet during the adult (Scheuber 2003) or juvenile (Scheuber et al. 2003) stages, even though diet can influence body size and the morphology of the calling apparatus. Thus as expected, the fine-scale temporal and spectral components of the male call (CPN, TN, intercall duration, CIPD and DF) that are a tightly integrated suite of traits under strong stabilizing selection (Brooks et al. 2005a; Bentsen et al. 2006) did not vary as a consequence of juvenile experience.

The more plastic traits are more often under directional sexual selection, although countervailing selection—such as viability selection—may place these traits under net stabilizing selection. These are the signal components most likely to advertise the signaler's “quality”; the big, bright, loud, odorous traits that we most associate with sexual selection. Although there is strong directional selection on male calling effort in *T. commodus* as females preferentially approach males that call at higher rates (Bentsen et al. 2006), how much a male spends calling each night (i.e., calling effort) is energetically costly (Hoback and Wagner 1997) such that males cannot maintain a high calling effort across many nights without being preyed on, dying from exhaustion, or senescing (Hunt et al. 2004; Zajitschek et al. 2009b). Calling effort has consistently been shown to vary with a suite of environmental cues ranging from diet (Hunt et al. 2004; Judge et al. 2008) to predation (Kolluru et al. 2002), and even inbreeding status (Drayton et al. 2010).

In general, calling effort for *T. commodus* follows a nonlinear trend with little calling soon after eclosion with a peak in mid life span and then some senescent decline in individuals who live long lives (Maklakov et al. 2008; Zajitschek et al. 2009a). In a previous study, we showed that the average investment a male makes in calling depends on the social environment he experiences as a juvenile (Kasumovic et al. 2011). Here we demonstrate that a complex interaction between two different social factors (the density and rate of calling males) affects this average by influencing how quickly calling effort declines in late adulthood (quadratic component; Table 3). Males exposed to higher calling rates as nymphs exhibited a more dramatic decline than males from either of the other two treatments, while males from the variable calling rate treatment maintained the highest calling effort late in life. Once again, it is variation in the calls experienced while immature, rather than binomial differences (e.g., presence/absence) that cues this shift in trait expression.

Apart from demonstrating differences in age-specific calling, our current results tell a more complete story than our previous analysis of the effect of the social environment on average daily calling effort (i.e., total lifetime calling effort divided by adult life span [Kasumovic et al. 2011]). The significant effect of density on life span (Kasumovic et al. 2011) coupled with the differences in how rapidly calling effort decreased as individuals from the different calling rate treatments aged (this study) both alter average daily calling effort. Our current analysis shows that calling effort trajectories did not differ until peak calling had been reached (around 30 days of adulthood) but that treatment differences in longevity and age-dependent decrease in calling effort are the likely causes of our earlier documented (Kasumovic et al. 2011) differences in average daily calling effort. Our results illustrate the pitfalls of averaging data from different time periods into a more crude analysis as information is lost.

Our earlier work showed that males exposed to cues suggesting an uncompetitive environment eclose more quickly (Kasumovic et al. 2011). These alterations may represent competition-mediated optimization of the trade-off between development rate and adult reproductive effort, with males willing to sacrifice phenotypic traits (e.g., larger size) by accelerating development and thereby exploiting less-competitive social conditions while they last. Although it is difficult to determine the fitness benefits associated with these developmental shifts as the social environment is dynamic, the changes in calling effort mirror developmental responses. Males in the variable calling rate treatment that took longer to eclose also took longer to reach their peak calling effort and maintained the highest calling effort late in life. This could increase attraction if males are attempting to avoid competition with current males that would decrease their calling effort by that point. In contrast, males that matured more quickly in the low and high calling rate treatments reached their peak earlier and maintained a higher average calling effort after the peak (Fig. 3), which may allow them to take advantage of the current lower competitive environment.

### Sexual selection and the social environment

Explaining the maintenance of variation in sexual signaling traits under strong stabilizing or directional selection remains a persistent problem in evolutionary biology (Barton and Turelli 1989; Barton and Keightley 2002; Blows and Hoffmann 2005) as strong directional selection or stabilizing selection depletes genetic variance (Hunt et al. 2007; McGuigan and Blows 2009) as predicted by theory (Bürger 2000; Johnson and Barton 2005). The relationship between selection and genetic variance is of particular concern in the study of mate choice because any erosion of variance in genetic quality will weaken the genetic benefit to individuals who choose to mate with high-quality individuals, potentially undermining the evolution of choice and questioning the value of the signaling trait as a cue of genetic quality. Although these results currently do not provide insight into how the social environment affects underlying genetic information, our results nevertheless have important consequences for the study of evolution as they provide insight into how social factors can affect sexually selected traits.

It is clear that the expression of sexually selected traits—especially plastic behavioral traits like calling—cannot be used as snapshots of individual “quality” in the way condition-dependent traits are usually thought (for a fuller treatment, see Lailvaux and Kasumovic 2011). This is because these results together with our previous developmental results (Kasumovic et al. 2011), we demonstrate that the perceived social environment affects both female choice and male sexual signals. As a result, adult quality cannot be stated without reference to the rearing context. Although the current results do not allow examination of fitness costs or benefits of the developmental and behavioral tactics that occur as a result of juvenile experience, it does not belie the fact that the social environment is introducing relevant variation in sexually selected traits through male experience, and potentially, female choice. The evolutionary consequences of the social environment must thus be carefully examined as socially cued anticipatory plasticity (Kasumovic and Brooks 2011) introduces variation in sexually selected traits in the same manner as does resource and nutrient abundance (i.e., condition).

The evidence that we present here does not necessarily contradict the currently ascendant idea that variation might best be maintained in condition-dependent traits that “capture” the underlying effects of large numbers of loci on condition (Rowe and Houle 1996; Tomkins et al. 2004). This idea of “genic capture” is inherently a notion of phenotypic plasticity. Although several authors have acknowledged that condition dependence is a form of phenotypic plasticity (Eraly et al. 2009; Saastamoinen et al. 2010; Stillwell et al. 2010), there has been no explicit theoretic integration of these areas of theory. Our findings suggest that other types of phenotypic plasticity, in this case socially cued anticipatory plasticity, might be equally germane to the questions of condition dependence and the maintenance of variation as other, more traditional sources of plasticity (i.e., dietary acquisition, physiological challenges, and aging) (e.g., Rode and Morrow 2009). This is especially true if there is a limit to which dietary acquisition alone can affect condition-dependent traits (e.g., Gosden and Chenoweth 2011). In addition to condition dependence, the expression of a sexually selected trait can vary plastically as a consequence of age (Preston et al. 2011), even interacting with condition (Hunt et al. 2004). Interestingly, our results demonstrate that juvenile social experience affects age-specific calling effort by shifting when individual's invest maximally in calling, suggesting somatic senescence may not be the only factor associated with age-specific changes in trait expression. Whether the rearing environment introduces only environmental variance or whether it releases genetic variance through indirect genetic effects and how this varies as a consequence remain interesting and important topics for future study.
